# Diagnostic measures in patients with severe insect sting reactions and elevated baseline serum tryptase levels 

**DOI:** 10.5414/ALX02524E

**Published:** 2024-08-19

**Authors:** Silvan Lange, Eva Oppel, Marius Winkler, Franziska Ruëff

**Affiliations:** Department of Dermatology and Allergy, LMU University Hospital, Munich, Germany

**Keywords:** insect venom allergy, anaphylaxis, venom immunotherapy, serum tryptase, mastocytosis, hereditary α-hypertryptasemia

## Abstract

Mastocytosis or an elevated basal serum tryptase (bST) level are known risk factors for patients with insect venom allergy. We report on 3 patients with a history of severe anaphylactic insect sting reactions who underwent a detailed workup for insect venom allergy before starting venom immunotherapy. In addition to insect venom sensitization, an elevated concentration of bST (15.5, 20.8, and 23.2 µg/L) was found in all cases. There was no evidence of mastocytosis in the skin (MIS). Further testing revealed hereditary α-hypertryptasemia (HαT) in 2 patients and a D816V mutation by liquid biopsy in 1 patient, which is a minor diagnostic criterion for indolent systemic mastocytosis. Even without iliac crest puncture, causes of elevated bST can be narrowed down with minimally invasive diagnostic measures. As this has practical implications, patients with elevated bST should always undergo further work-up to determine the cause of this abnormal finding.

## Introduction 

In adults, on average, ¼ (18 – 42%) of Hymenoptera venom (HV)-induced allergic reactions are severe and may result in anaphylactic shock (Ring and Messmer grade III) [1] or the need for cardiopulmonary resuscitation (grade IV) [2]. The strongest risk factor for severe reactions is mastocytosis [3]. However, even without a formal diagnosis of mastocytosis, elevated basal serum tryptase (bST) is a risk factor for severe reactions [4]. 

Screening for mastocytosis (skin examination, bST measurement) is recommended in adult patients with suspected HV allergy at first presentation [4]. The combination of elevated bST without mastocytosis in the skin (MIS) is not uncommon and raises the question of how to proceed with further diagnostic procedures and what therapeutic consequences may result. 

We report on 3 patients with elevated bST and a history of systemic allergic reaction (SAR) following an insect sting who were seen in our outpatient clinic for diagnostic workup and initiation of venom immunotherapy (VIT). 

## Case reports 

The main clinical data and test results of the 3 patients are shown in Table 1. 

Skin and in vitro tests showed HV sensitization in all cases. In addition to repeated bST testing (ImmunoCAP, Thermo Fisher Scientific, Freiburg, Germany), testing for hereditary α-hypertryptasemia (HαT) was performed by mutation analysis of the *TPSAB1* gene and a so-called liquid biopsy to detect a D816V mutation. These tests showed that 2 of our patients had HαT; 1 patient fulfilled a minor diagnostic criterion for indolent systemic mastocytosis (SM) without concomitant HαT. 

All patients had a history of severe symptoms after an insect sting (Ring and Messmer grade III), and 1 patient developed repeated anaphylactic reactions during VIT (Ring and Messmer grade II), which resolved only after concomitant omalizumab treatment and after increasing the maintenance dose to 200 µg of bee venom. The patient did not agree to a sting challenge test while tolerating maintenance VIT. Despite ongoing maintenance therapy with 100 µg of wasp venom, patient 2 experienced a grade II anaphylactic reaction following a wasp field sting. After increasing the dose of wasp venom to 200 µg, the patient was clearly protected according to the results of a further sting provocation. Only patient 1 had an uneventful VIT; the establishment of clinical protection could not be demonstrated as this patient did not wish to undergo a sting challenge. However, the patient reported 3 field stings since the start of VIT, all of which were well tolerated with no serious reactions. 

## Discussion 

The HV-allergic patients presented here were noteworthy in several respects: all patients initially experienced severe sting reactions, 1 patient had repeated systemic side effects during VIT, and 1 patient initially proved unprotected at the standard dose during sting provocation. In all 3 patients, bST was elevated at initial diagnosis. However, in 1 patient bST was above the 95^th^ percentile, but – in contrast to the other 2 patients – it was not > 20 µg/L, thus not fulfilling the corresponding minor diagnostic criterion of SM (Table 2). Genetic testing revealed that 2 of our patients had HαT; one patient was positive for a minor diagnostic criterion for indolent SM but did not have concomitant HαT. 

It should not be assumed that mastocytosis is always associated with elevated bST. A bST level within the 95^th^ percentile does not exclude mastocytosis. In a larger case series, ~ 40% of patients with indolent SM had a bST level of less than 20 µg/L, thus failing to meet a minor diagnostic criterion for indolent SM. Of these 40%, approximately half, i.e., 20% of all patients with SM, had a bST level of < 11.4 µg/L (corresponding to the 95^th^ percentile) [5]. For the diagnosis and classification of mastocytosis, we refer to a recent consensus paper [6]. 

In clinical practice, there is often uncertainty about the diagnostic procedure to follow once an elevated bST has been identified. Firstly, if the bST is elevated, the analysis should be repeated and the skin inspected for signs of MIS. In our patients, skin examinations were negative. If MIS is present in adult patients, it is highly likely that SM is also present [5]. In patients with insect venom allergy and SM, the latter is typically an indolent form of SM. 

If patients do not present with MIS (caution: skin changes can be very subtle and easily overlooked!) and SM is suspected, further diagnostic workup is indicated to confirm or rule out SM. Less invasive methods can be used to narrow down the cause of elevated bST in some patients. If a D816V mutation can be detected by blood testing, the diagnosis of SM is more likely. To confirm the diagnosis, it may be necessary to check other parameters (Table 2), usually including an iliac crest puncture [6]. However, patient 3 did not agree to have the recommended examinations. 

Possible practical implications for patients with insect venom allergy in the presence of SM include treatment modifications, possibly including the use of an increased maintenance dose starting from the beginning of VIT and continued throughout VIT [7]. In addition, patients with SM have an increased risk of pathological bone density findings, most commonly osteopenia and osteoporosis, and less commonly osteosclerosis or osteolysis [8]. Further molecular genetic classification of SM is also important, as the long-term prognosis depends on the type of mutation detected, and patients should be enrolled in an appropriate follow-up program. 

The detection or exclusion of mastocytosis is not only of academic value but also of immediate practical value for the patients concerned. However, if another cause of elevated bST was found (as in patients 1 and 2) and blood tests for the D816V mutation were negative, we believe that those patients do not need an iliac crest puncture. 

HαT as a possible cause of elevated bST was described only a few years ago [9]. In such cases, there is a duplication or triplication of the *TPSAB1* gene, which codes for α-tryptase, leading to a subsequent increase in bST. Patients with HαT also have an increased risk of anaphylaxis [10], which should be subject to a rigorous workup, as positive findings (such as insect venom allergy) have practical implications. In principle, patients with SM may also have HαT. This combination may increase the risk of anaphylaxis. In patients with SM, bST levels are thought to reflect mast cell proliferation. If HαT is also present, the bST concentration should be interpreted differently. 

It should be noted that elevated bST may have causes other than mastocytosis or HαT, most notably myeloproliferative disorders in which bST is typically massively elevated. To rule out such conditions, and if common causes cannot be found, further search for the underlying cause of the elevated bST is indicated. 

Ultimately, the diagnosis of SM can only be confirmed or excluded by an iliac crest puncture. However, many patients are reluctant to undergo this invasive procedure without a plausible explanation of the consequences for them. If indolent SM cannot be ruled out, we treat patients with insect venom allergy as if SM had been confirmed, i.e., VIT is adjusted accordingly in terms of target dose and duration of therapy, and routine care is provided as for SM patients. 

## Essential sentence 

Even without an iliac crest puncture, the causes of elevated bST can be narrowed down by minimally invasive diagnostic measures. As positive results have practical consequences, particularly in the case of insect venom allergies, further diagnostic measures should be taken in the event of an elevated bST. 

## Authors’ contributions 

Hereby, we confirm that all authors made substantial contributions to the conception or design of the work; the acquisition, analysis, and interpretation of data; and drafting the work and revising it critically. All authors approved the final version. 

## Funding 

The authors received no funding for this case report. 

## Conflict of interest 

FR has participated in advisory boards for ALK-Abelló Arzneimittel GmbH, Blueprint medicines (Germany), and received payments for lectures from ALK-Abelló Arzneimittel GmbH Novartis, and ThermoFisher. MW received payments for lectures from ALK-Abelló Arzneimittel GmbH. The other authors declare that there are no conflicts of interest in connection with this case report. 

**Table 1.** Characterization of the patients.

**Patient**		**1**	**2**	**3**
Age at time of first presentation and biological sex		47 yrs, male	46 yrs, female	34 yrs, female
Sting history: Type of insect, severity of sting reaction (Ring and Messmer)		Wasp, repeated SAR (grade III)	Wasp, SAR grade III	Bee, SAR grade III
Insect venom-specific IgE concentration at first presentation kUA/L (CAP class)	Bee	0.91 (2)	0.15 (0)	6.22 (3)
	Vespula	0.55 (1)	9.81 (3)	0.05 (0)
Lowest venom concentration resulting in a positive skin prick test	Bee	Negative up to 100 µg/mL	Negative up to 300 µg/mL	n.d.
	Vespula	Negative up to 100 µg/mL	Negative up to 300 µg/mL	n.d.
Intradermal test 1 μg/mL	Bee	Negative	n.d.	n.d.
	Vespula	Positive	n.d.	n.d.
bST (µg/L)		20.8	23.2	15.5
Venom dose for initial treatment		200 µg bee venom and 100 µg Vespula venom	100 µg Vespula venom	200 µg bee venom
Symptoms during VIT		No SAR	No SAR	Repeated SAR (grade II) good tolerance of dosing-up while receiving omalizumab (for 5 months)
Reactions after sting provocation/field sting		n.d.	SAR grade II following a field sting No SAR following VIT dose increase	No SAR
bST (µg/L) during VIT		20.2	17.2	16.3
TPSAB1 gene mutation		Germline duplication	Germline triplication	Absent
KIT mutation (D816V)		Absent	Absent	Present
Bone densitometry findings		Normal	Mild osteopenia	n.d.

SAR = systemic allergic reaction; n.d. = not done; bST = basal serum tryptase; VIT = venom immunotherapy.

**Table 2.** Criteria for the diagnosis of systemic mastocytosis [6].

Major criterion	Multifocal dense infiltrates of mast cells in bone marrow biopsies and/or in sections of other extracutaneous organs
Minor criteria	≥ 25% of all mast cells in sections of bone marrow or other extracutaneous organs are atypical, e.g., spindle-shaped
	KIT-activating KIT point mutation at codon 816 in the bone marrow or another extracutaneous organ
	CD2 and/or CD25 and/or CD30 expression on mast cells
	Basal tryptase in serum > 20 µg/L
If at least 1 major and 1 minor criterion or 3 minor criteria are met, the diagnosis of a systemic mastocytosis is confirmed.	
